# The Cortical Response Evoked by Robotic Wrist Perturbations Reflects Level of Proprioceptive Impairment After Stroke

**DOI:** 10.3389/fnhum.2021.695366

**Published:** 2021-11-09

**Authors:** Joost van Kordelaar, Mark van de Ruit, Teodoro Solis-Escalante, Leo A. M. Aerden, Carel G. M. Meskers, Erwin E. H. van Wegen, Alfred C. Schouten, Gert Kwakkel, Frans C. T. van der Helm

**Affiliations:** ^1^Department of Biomechanical Engineering, Delft University of Technology, Delft, Netherlands; ^2^Department of Rehabilitation, Donders Institute for Brain, Cognition and Behavior, Radboud University Medical Center, Nijmegen, Netherlands; ^3^Department of Neurology, Reinier de Graaf Hospital, Delft, Netherlands; ^4^Department of Rehabilitation Medicine, Amsterdam Neuroscience and Amsterdam Movement Sciences, Amsterdam University Medical Center, Amsterdam, Netherlands; ^5^Department of Biomedical Engineering, University of Twente, Enschede, Netherlands

**Keywords:** stroke, electroencephalography, afferent pathways, proprioception, motor function, prognosis

## Abstract

**Background:** Proprioception is important for regaining motor function in the paretic upper extremity after stroke. However, clinical assessments of proprioception are subjective and require verbal responses from the patient to applied proprioceptive stimuli. Cortical responses evoked by robotic wrist perturbations and measured by electroencephalography (EEG) may be an objective method to support current clinical assessments of proprioception.

**Objective:** To establish whether evoked cortical responses reflect proprioceptive deficits as assessed by clinical scales and whether they predict upper extremity motor function at 26 weeks after stroke.

**Methods:** Thirty-one patients with stroke were included. In week 1, 3, 5, 12, and 26 after stroke, the upper extremity sections of the Erasmus modified Nottingham Sensory Assessment (EmNSA-UE) and the Fugl-Meyer Motor Assessment (FM-UE) and the EEG responses (64 channels) to robotic wrist perturbations were measured. The extent to which proprioceptive input was conveyed to the affected hemisphere was estimated by the signal-to-noise ratio (SNR) of the evoked response. The relationships between SNR and EmNSA-UE as well as SNR and time after stroke were investigated using linear regression. Receiver-operating-characteristic curves were used to compare the predictive values of SNR and EmNSA-UE for predicting whether patients regained some selective motor control (FM-UE > 22) or whether they could only move their paretic upper extremity within basic limb synergies (FM-UE ≤ 22) at 26 weeks after stroke.

**Results:** Patients (*N* = 7) with impaired proprioception (EmNSA-UE proprioception score < 8) had significantly smaller SNR than patients with unimpaired proprioception (*N* = 24) [EmNSA-UE proprioception score = 8, *t*(29) = 2.36, *p* = 0.03]. No significant effect of time after stroke on SNR was observed. Furthermore, there was no significant difference in the predictive value between EmNSA-UE and SNR for predicting motor function at 26 weeks after stroke.

**Conclusion:** The SNR of the evoked cortical response does not significantly change as a function of time after stroke and differs between patients with clinically assessed impaired and unimpaired proprioception, suggesting that SNR reflects persistent damage to proprioceptive pathways. A similar predictive value with respect to EmNSA-UE suggests that SNR may be used as an objective predictor next to clinical sensory assessments for predicting motor function at 26 weeks after stroke.

## Introduction

It is estimated that each year 6.8 million people worldwide suffer from an ischemic stroke (Feigin et al., 2015). Almost 80% of these patients show paresis, i.e., weakness, in one of the upper extremities in the first three weeks after stroke (Lawrence et al., 2001). Improvements in motor function are mainly observed in the first three months after stroke, however, the rate of this time-dependent recovery differs greatly between subjects (van der Vliet et al., 2020).

Spontaneous neurobiological recovery is generally considered to be the main driver of motor recovery early after stroke (Krakauer, 2005; Langhorne et al., 2011), which follows a typical recovery pattern in which most improvements occur within the first ten weeks after stroke (van der Vliet et al., 2020). However, the exact neurophysiological mechanisms that contribute to spontaneous neurobiological recovery are still poorly understood. It is particularly unclear why spontaneous neurobiological recovery differs greatly between patients (Boyd et al., 2017; Ward, 2017; van der Vliet et al., 2020).

Recent prognostic studies suggest that motor function at 26 weeks after stroke is, to a large extent, defined by the integrity of the corticospinal tract (Stinear, 2017). These studies assessed the integrity of descending motor pathways by evaluating the ability to extend the fingers of the paretic hand (Stinear et al., 2007; Nijland et al., 2010) or by measuring the motor evoked potential induced by transcranial magnetic stimulation of the primary motor cortex (van Kuijk et al., 2009; Stinear et al., 2017; Hoonhorst et al., 2018). Next to integrity of the *descending* pathways, integrity of *ascending* somatosensory pathways seems to be an important predictor for motor function after stroke. In particular the proprioceptive system seems to play a crucial role in regaining control over motor tasks (Vidoni and Boyd, 2009; Simo et al., 2014; Zandvliet et al., 2020a,b). To assess the integrity of ascending pathways, previous studies used the upper extremity section of the Erasmus modified Nottingham Sensory Assessment (EmNSA-UE) (Winters et al., 2016; Zandvliet et al., 2020a). The EmNSA-UE provides separate subscores for proprioception and tactile function. However, performance on the test is represented on a 3-point scale for each joint and therefore the resolution of this scale is poor (Contu et al., 2017). In addition, the EmNSA-UE requires patients to verbally respond to sensory stimuli applied by the experimenter. As such the EmNSA-UE might not be suitable in patients with aphasia or attention deficits. Other prediction studies have partly overcome these limitations of the EmNSA-UE by using the magnitude or latency of the somatosensory evoked potential (SSEP) as measured in the electroencephalogram (EEG) to predict motor function after stroke (Feys et al., 2000; Al-Rawi et al., 2009). However, a recent study concludes that the reliability of SSEP is poor and the location of the SSEP is variable between and within subjects (Kalogianni et al., 2018). In addition, SSEP reflects the evoked cortical response to a mixture of sensory inputs when evoked by electrical stimulation of the median nerve in the paretic upper extremity. As a consequence, SSEP indicates the overall integrity of all afferent somatosensory modalities together, including tactile and thermal functions and pain, rather than proprioception alone. Therefore, there is a need for objective metrics that reliably quantify proprioceptive function without the need for patients to verbally respond to somatosensory stimuli.

In the present study we utilized a robot to induce passive wrist flexion and extension movements. We aimed to stimulate both type Ia and type II afferents, which detect fast and slow changes in muscle stretch, respectively (Macefield and Knellwolf, 2018). Both types of afferents ascend within the dorsal column tract and project onto dorsal column nuclei in the brainstem. These nuclei convey proprioceptive information contralaterally to the ventroposterior superior nucleus of the thalamus, which mainly projects to the primary somatosensory cortex (Delhaye et al., 2018). We used multichannel EEG to measure the evoked cortical response, i.e., the proprioceptive information arriving at the cortex, in response to the passive wrist movements imposed by the robot. We assumed that the magnitude of the evoked cortical response relative to ongoing brain activity, as quantified by the signal-to-noise ratio (SNR), reflects the extent to which proprioceptive input was conveyed to the brain (Vlaar et al., 2017).

A previous study from our group showed that SNR is significantly smaller in patients with severe proprioceptive deficits as compared to patients with no or only mild proprioceptive deficits and healthy subjects (Vlaar et al., 2017). The present study capitalizes on this study by pursuing the following aims. First, we aimed to assess whether SNR follows the typical recovery pattern that reflects spontaneous neurobiological recovery in which improvements occur mainly during the first three months after stroke. Second, we aimed to establish whether SNR is different for patients with impaired and unimpaired proprioception, as measured with the proprioception score of the upper extremity section of the EmNSA-UE, in the first 26 weeks after stroke. Third, we aimed to determine whether motor function at 26 weeks after stroke can be more accurately predicted with the SNR as compared with the proprioceptive subsection and the total score of the EmNSA-UE measured within the first three weeks after stroke.

We hypothesized that SNR follows the typical pattern of recovery in which most improvements are observed within ten weeks after stroke. We also hypothesized that SNR reflects proprioceptive integrity and is therefore larger for patients with unimpaired proprioceptive function as compared to patients with impaired proprioception as measured with EmNSA-UE. Lastly, as SNR is an objective measure on a continuous scale, we hypothesized that it can more accurately predict motor recovery after stroke as compared to EmNSA-UE measured within the first three weeks after stroke.

## Materials and Methods

### Recruitment

Patients were recruited for this observational study in 6 hospitals in The Netherlands. The study was approved by the Medical Ethical Reviewing Committee of the Amsterdam University Medical Center, location VU University Medical Center (registration number 2014.140) and carried out in accordance with The Code of Ethics of The World Medical Association (2020).

### Participants

Patients were screened for inclusion into the present study within 3 weeks, but preferably within one week after stroke. Patients were included when (1) they had a first-ever stroke in an area supplied by the anterior, medial and/or posterior arteries verified by CT and/or MRI scan, (2) they had an upper extremity paresis as indicated by a score of 1 or larger on the National Institutes of Health Stroke Scale (NIHSS), (3) they were 18 years or older, (4) they had no severe cognitive impairments as indicated by a Mini Mental State Examination (MMSE) score of 18 or larger and (5) they were able to sit without support. Patients were excluded when they had (1) previously existing pathological neurological conditions, (2) previously existing orthopedic limitations that would affect the results, and/or (3) botuline-toxine injections or medication that may influence upper extremity function in the previous three months. All patients gave written informed consent before participating in the study.

### Clinical Assessments

All patients participated in a series of clinical measurements conducted weekly until week 5 after stroke and subsequently in week 8, 12, and 26. For this study scores from the EmNSA-UE and the upper extremity section of the Fugl-Meyer Motor Assessment (FM-UE) from week 1, 3, 5, 12, and 26 were used.

The EmNSA-UE evaluates two modalities of sensory function, namely, tactile function and proprioception. For tactile function a maximum score of 32 can be obtained. The maximum score for proprioception is 8 leading to a maximum total score of 40 for the entire assessment. Two-point discrimination was not assessed as this is an unreliable test item (Stolk-Hornsveld et al., 2006). In the present study the score of the proprioceptive subsection (EmNSA-UE_p_) and the total score of the EmNSA-UE (EmNSA-UE_t_) are used for further analysis.

The FM-UE is a reliable and valid test based on the sequential stages of motor recovery (Fugl-Meyer et al., 1975; Gladstone et al., 2002). It assesses reflexes, basic limb synergies and hand function of the paretic upper extremity. Each item is scored on a three- point scale leading to a maximum score of 66 points.

### Electroencephalography Measurements

Next to the clinical assessments, patients participated in a series of EEG measurements conducted in week 1, 3, 5, 12, and 26 after stroke in a specially equipped measurement van (Volkswagen Crafter, Wolfsburg, Germany). The procedure for these measurements has been previously described in detail elsewhere (Vlaar et al., 2017). Briefly, scalp potentials were collected using 64 Ag/AgCl electrodes arranged according to a subset of the extended 10/20 system. A ground electrode was placed on the left mastoid process. All signals were amplified using a Refa amplifier (TMSi, Oldenzaal, The Netherlands) sampled at 2048 Hz and using anti-aliasing hardware filters. During the entire measurement, patients sat in a comfortable wheelchair (Ibis, Sunrise Medical Incorporated, Fresno, CA, United States). The forearm and hand of the paretic upper extremity were strapped to the handle of a robot (“Wristalyzer,” MOOG, Nieuw-Vennep, The Netherlands), such that the elbow was in 90 degrees flexion and the wrist joint was aligned with the rotation axis of the robot. The hand was strapped to the handle with the fingers extended and the design of the handle prevented the fingers from holding the edge, thereby preventing an active grip by the patients. Patients were instructed to sit quietly and to keep their arm, wrist and hand relaxed. A computer screen approximately one meter in front of the patients showed a cross-hair which served as a gaze fixation target during data collection.

**FIGURE 1:**
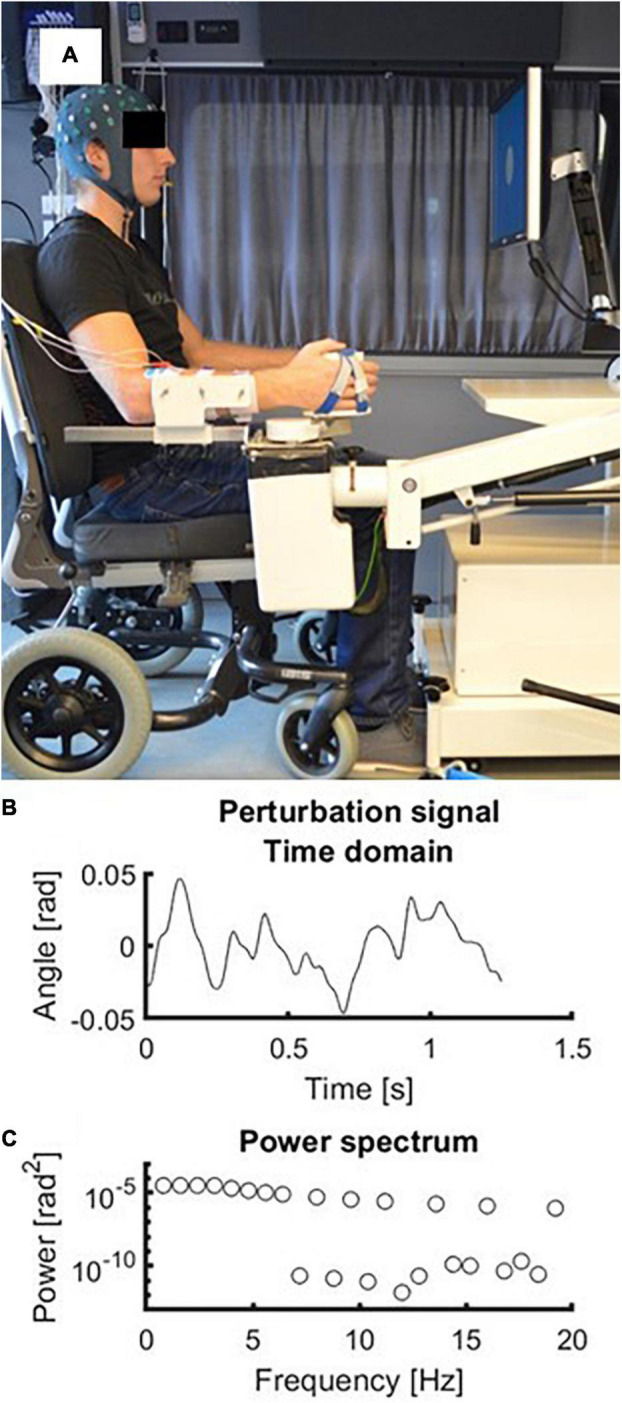
Experimental setup. **(A)** Picture of the experimental setup with a subject wearing the EEG cap and with his forearm strapped in the handle of the robot. **(B)** Time domain representation of the perturbation signal. Zero radians represents the neutral position of the wrist at 0.35 rad (i.e., 20 degrees) flexion. **(C)** Frequency domain representation of the perturbation signal. Frequencies above 4 Hz had decreasing amplitudes (−20 dB/dec).

During each trial the wrist angle was continuously moved by the robot in the horizontal plane following a multisine perturbation signal. The perturbation signal consisted of the sum of multiple sines which varied in frequency and phase. The period of the perturbation signal was 1.25 s and the root mean square excursion of the signal was 0.02 rad. The signal contained power at the following frequencies: 0.8, 1.6, 2.4, 3.2, 4.0, 4.8, 5.6, 6.4, 8.0, 9.6, 11.2, 13.6, 16.0, and 19.2 Hz. The perturbation was imposed to a relaxed position of the wrist, which corresponded to a slight wrist flexion of 0.35 rad. The signal was repeated 10 times within a trial, leading to a trial duration of 12.5 s. During each measurement 20 trials were recorded. Therefore, there were 200 periods of the perturbation signal available for analysis. The experimental setup including the perturbation signal is shown in Figure 1.

### Electroencephalography Analysis

Analysis of all EEG data was conducted with MATLAB (Version R2018b; The Mathworks, Natick, MA, United States) and Fieldtrip (Oostenveld et al., 2011). Raw EEG data was filtered using a fourth-order recursive Butterworth band-pass filter between 0.8 and 120 Hz. Line noise and its first harmonic were removed with a fourth-order recursive Butterworth bandstop filter between 49 and 51 Hz and between 99 and 101 Hz. EEG data was then segmented into epochs of 1.25 s (i.e., the length of the perturbation). The first 2 epochs of each trial were removed to avoid transient effects after the start of the perturbation resulting into a total of 160 (i.e., 20 trials times 8 epochs) epochs per measurement.

Based on visual inspection disconnected channels or channels with a severe amount of noise (amplitude > 50 μV) were deleted. Similarly, based on visual inspection all epochs that were severely contaminated with muscle activation artefacts were removed from the data. All remaining channels were re-referenced to the common average. To separate brain activity from eye blinks and remaining sources of noise Infomax independent component analysis (runica.m) was conducted (Makeig et al., 1996). An equivalent current dipole was fitted on the scalp distribution of each independent component. Components were removed if (1) the residual variance of the associated dipole was above 15%), (2) they represented muscle activation (increasing power spectrum between 15 and 40 Hz), (3) their scalp distribution corresponded with only one electrode or (4) they represented eye blinks [based on frontal scalp distribution and typical eye blink profile in the time domain representation (Hoffmann and Falkenstein, 2008)]. All retained components were projected back to electrode level to create artefact-free EEG.

The steady-state response (SSR) for each electrode was defined as the average cortical response to the periodic wrist perturbations and it was estimated by taking the mean EEG signal x(k) across epochs (e):


(1) $$\text{SSR}\,\left(\text{k}\right)=\frac{1}{E}\,\sum_{e=1}^{E}x_{e}\,\left(k\right)$$

To determine the SNR of each electrode we computed the signal power S as the sum of squared SSR across samples k within each epoch:


(2) $$S=\sum_{k=1}^{K}\mathrm{SSR}\,{\left(k\right)}^{2}$$

Then, power of the noise N for each electrode was obtained by taking the mean variance of the EEG signal across epochs and samples:


(3) $$\text{N}=\sum_{k=1}^{K}\frac{1}{E-1}\,\sum_{e=1}^{E}{\left(x_{e}\,\left(k\right)-\mathrm{SSR}\,\left(k\right)\right)}^{2}$$

Finally, the SNR for each electrode was obtained by dividing S by N and transforming to decibel (dB):


(4) $${\text{SNR}}_{\text{electrode}}=10\,{\text{log}}_{10}\,\left(\frac{\text{S}}{\text{N}}\right)$$

A previous cross-sectional study from our group showed that healthy subjects and patients with no or only mild proprioceptive impairments show significantly higher SNR in the hemisphere contralateral to the perturbed wrist, compared to patients with severe proprioceptive impairment. SNR in de ipsilateral hemisphere did not differ significantly between groups (Vlaar et al., 2017). Therefore, we decided to adopt the same region-of-interest (ROI) as in the study of Vlaar et al. (2017) to determine the mean SNR in the hemisphere contralateral to the perturbed wrist, which was the affected hemisphere in this study.

For patients with a paresis in the right upper extremity, this ROI consisted of the following odd electrodes at the left side of the head: F1, F3, F5, FC1, FC3, FC5, C1, C3, C5, CP1, CP3, CP5, P1, P3, and P5. For patients with a paresis in the left upper extremity their even counterparts at the right side of the head were included: F2, F4, F6, FC2, FC4, FC6, C2, C4, C6, CP2, CP4, CP6, P2, P4, and P6. This ROI covered parts of the frontal and parietal cortex. The mean SNR_*elec*__*trode*_ in dB within this region-of-interest was calculated and this SNR value was used for subsequent statistical analysis (Vlaar et al., 2017).

### Dichotomization

Patients were allocated into one of two proprioception groups. Patients with an initial maximal EmNSA-UE_p_ score of 8 points were allocated to the “unimpaired proprioception group” and patients with an initial EmNSA-UE_p_ score smaller than 8 were allocated into the “impaired proprioception group.” Furthermore, FM-UE scores at 26 weeks after stroke were dichotomized. Based on a recent study (Hoonhorst et al., 2015), patients were considered “recoverers” if they had regained some selective motor control, as indicated by a score larger than 22 on the FM-UE at 26 weeks after stroke. Patients were considered “non-recoverers” if they could only move their paretic upper extremity within basic limb synergies, as indicated by a score smaller than or equal to 22 on the FM-UE at 26 weeks after stroke.

### Statistical Analysis

All statistics were conducted in R (R Core Team, 2020). A linear mixed model was used to investigate the effect of proprioception group *G* (i.e., unimpaired/impaired) and time after stroke t on SNR:


(5) $${\mathrm{SNR}}_{i,t}=\left(\beta_{0}+b_{0_{i}}\right)+\beta_{1}\cdot G+\beta_{2}\cdot t+\beta_{3}\cdot G\cdot t+\varepsilon_{i,t},$$

where SNR_i,t_ is *SNR* for the i^th^ patient at time point t after stroke. β_0_ is a fixed offset, β_1_ is a fixed main effect for proprioception group G, β_2_ is a fixed main effect for time point after stroke, β_3_ is a fixed interaction effect for group G times time after stroke t, b_0_i_ is a random intercept for each patient and ε_i,t_ is the error for the i^th^ patient at time point t after stroke. The model was fitted using the restricted maximum likelihood (REML) approach (West et al., 2014).

Lastly, receiver operating characteristic (ROC) curves (R function *roc*) were used to determine the predictive value of the SNR, EmNSA-UE_p_ and EmNSA-UE_t_ (Robin et al., 2011). For each measure, the value at the first measurement was taken and an ROC curve was determined to investigate to what extent this value predicts whether patients ended up in the “recoverer” or “non-recoverer” group. The area under the ROC curve (AUC) was used as a measure of the predictive value.

We tested whether the AUC of all three ROC curves differed significantly from one another using the DeLong approach as implemented in the R function *roc.test* (DeLong et al., 1988; Robin et al., 2011). Critical alpha level for all statistical analyses was set two-tailed at 0.05. Corrections for multiple testing were done using the false recovery rate procedure (Curran-Everett, 2000).

## Results

**FIGURE 2:**
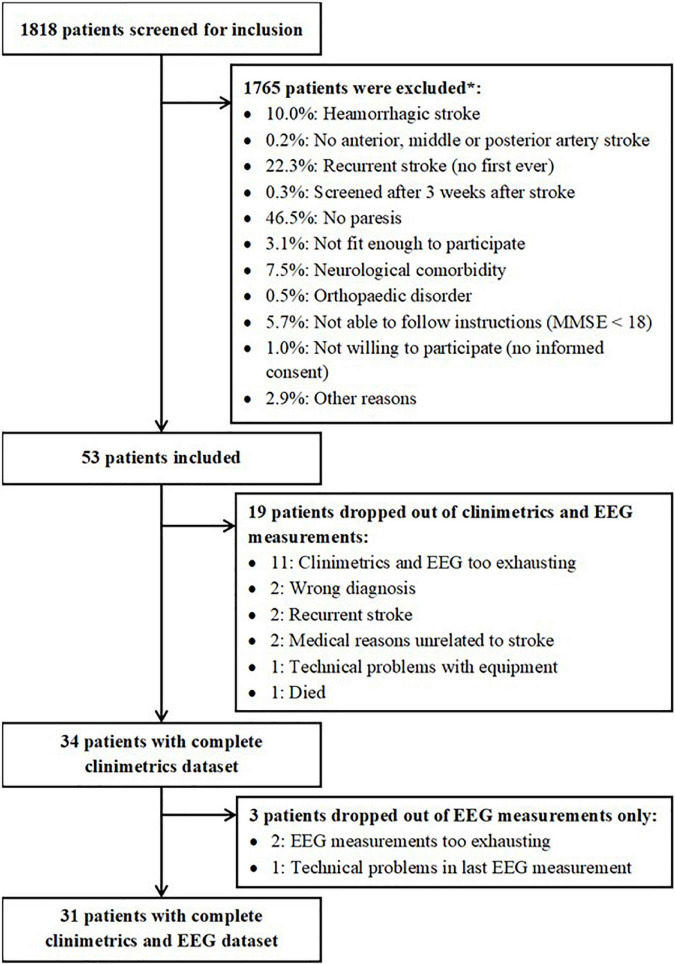
Overview of patients who were included and excluded for the present study. *Estimations based on inclusion/exclusion information from one of the participating centers.

Figure 2 depicts the patient inclusion flow-chart, which was designed according to the STROBE checklist for observational studies (von Elm et al., 2008). This flowchart shows that 1818 patients were screened for inclusion. Eventually, fifty-three patients were included for the present study. Thirty-four patients had complete clinimetric data, of whom thirty-one patients also had complete EEG data up to 26 weeks after stroke. Baseline characteristics of these patients are reported in Table 1. Individual patient data at each timepoint is provided in Supplementary Table 1.

**TABLE 1:** Baseline characteristics of patients included in the study.

**Characteristic**	**Values sample^1^ (*N* = 34)**	**Values subsample^2^ (*N* = 31)**
Sex (male/female), N	20/14	19/12
Age (years)^*^	70.6 ± 11.6	69.5 ± 11.5
EmNSA-UE total score (max = 40)^**^	37 (34 – 40)	37 (33.50 – 40)
EmNSA-UE proprioception score (max = 8)^**^	8 (8 – 8)	8 (7.75 – 8)
FM-UE (max = 66)^**^	16.5 (7 – 41.25)	18 (7 – 40.5)

N Number of patients, EmNSA-UE upper extremity section of the ErasmusMC modified Nottingham Sensory Assessment, FM-UE upper extremity section of the Fugl-Meyer Motor Assessment. ^1^Characteristics of all patients with complete clinimetric data but incomplete EEG data ^2^Characteristics of a subset of patients with complete clinimetric data and complete EEG data *Mean and standard deviation **Median and interquartile range.

### Effect of Proprioceptive Impairment and Time After Stroke on Signal-to-Noise Ratio

**FIGURE 3:**
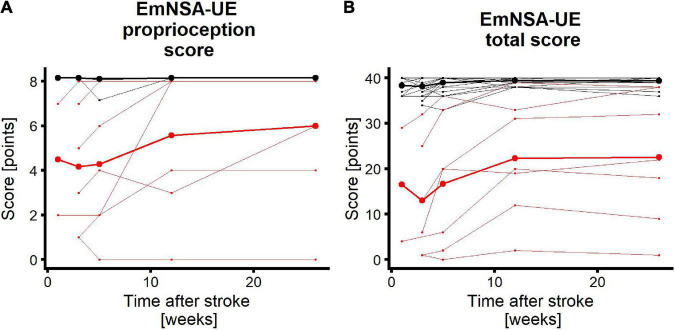
Longitudinal recovery profiles. Red/black lines represent patients with impaired/unimpaired proprioceptive function within 3 weeks after stroke. Each thin line represents a single patient. Thick lines are mean recovery profiles within the two groups of patients. **(A)** EmNSA-UE proprioception score (EmNSA-UE_p_) for all patients who participated in the clinimetric and EEG measurements up to week 26 after stroke. Maximum score is 8 points. Maximum value for unimpaired patients were added with 0.15 for plotting purposes. **(B)** EmNSA-UE total score (EmNSA-UE_t_) for the same patients; maximum score is 40 points. Note that patients with unimpaired proprioceptive function largely overlap.

Figure 3 shows the recovery profiles of EmNSA-UE_p_ and EmNSA-UE_t_ for the 31 patients with complete clinimetrics and EEG data. Seven patients were labeled as having impaired proprioception based on EmNSA-UE_p_ within 3 weeks after stroke, 24 patients had unimpaired proprioception.

**FIGURE 4:**
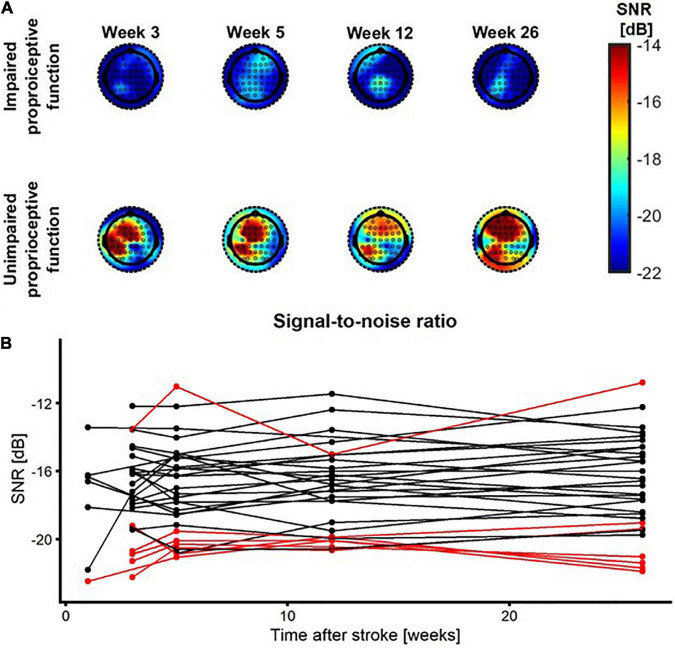
SNR as a function of time after stroke. **(A)** SNR topoplots for one patient with impaired proprioception (top row) and for one patient with unimpaired proprioception (bottom row) at week 1, 3, 5, 12, and 26 after stroke. **(B)** SNR as a function of time after stroke. Red/black lines represent patients with impaired/unimpaired proprioception at baseline, respectively.

Figure 4 shows the SNR topoplots for one patient with impaired and one patient with unimpaired proprioceptive function. Based on these topoplots it is clear that there is a large region with increased SNR in the patient with unimpaired proprioceptive function, whereas in the patient with impaired proprioception SNR is substantially lower across all electrodes. The SNR at each time point after stroke is plotted for each patient and each condition. Almost all patients with impaired proprioceptive function show the lowest values for SNR of the entire sample of patients at all timepoints after stroke. There was only one outlier, which included one patient with impaired proprioceptive function who showed considerable larger values of SNR than the other patients with impaired proprioceptive function. The fit of the linear mixed model in eq. 5 indicated that there was a significant main effect of group on the magnitude of SNR [β = 3.8, *t*(29) = 2.36, *p* = 0.03]. There was no significant main effect of time after stroke or interaction effect between time and group on the magnitude of SNR (*p* > 0.16).

### Predictive Value of Signal-to-Noise Ratio as Compared to EmNSA-UE_p_ and EmNSA-UE_t_

**FIGURE 5:**
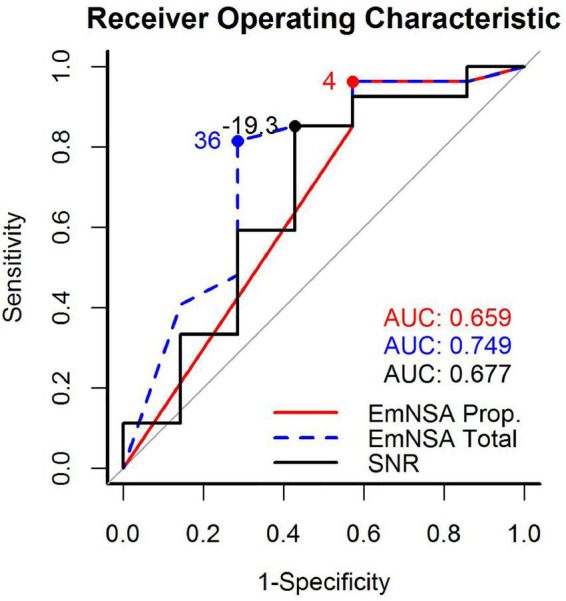
Receiver operating characteristic (ROC) curves for the proprioception score of the EmNSA-UE, the total score of the EmNSA-UE and SNR. The area under curve (AUC) for each ROC curve as well as the optimal cut-off score to distinguish between recoverers and non-recoverers are given as text inset.

**FIGURE 6:**
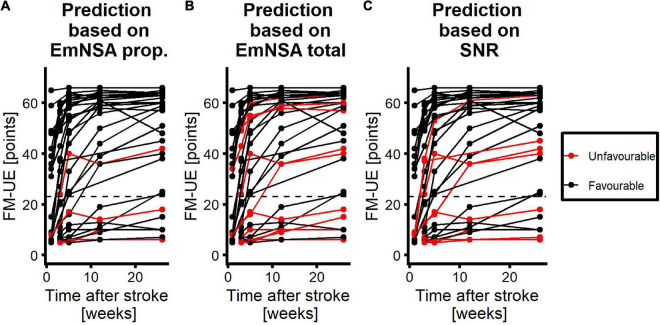
Performance of each predictor to predict motor function at 26 weeks after stroke. Red curves represent patients with unfavorable prognosis, i.e., they are expected to have an FM-UE score smaller than or equal to 22 at 26 weeks after stroke. Black curves represent patients with favorable prognosis for motor function at 26 weeks, i.e., they are expected to have an FM-UE score larger than 22 at 26 weeks after stroke. **(A)** Performance of the proprioception score of the EmNSA-UE. **(B)** Performance of the total score of the EmNSA-UE. **(C)** Performance of SNR. Dashed line represents FM-UE = 23 points.

The ROC curves for SNR, EmNSA-UE_p_ and EmNSA-UE_t_ to predict motor function at 26 weeks after stroke are given in Figure 5. The ROC curves are based on the 34 patients with a complete clinimetric dataset and at least one EEG measurement within 3 weeks after stroke. Based on these ROC curves, the optimal cut-off score for each predictor was determined that led to the largest AUC, representing the highest sensitivity and specificity. Figure 6 shows the FM-UE data for each patient as a function of time after stroke. It also indicates for which FM-UE recovery profiles a correct prediction was achieved by each predictor. Based on Figure 5, the EmNSA-UE_p_ (cut-off score = 4 out of 8, AUC = 0.659) has the highest sensitivity (largest number of true positives), yet poorest specificity (largest number of false positives). In contrast, EmNSA-UE_t_ (cut-off score = 36 out of 40, AUC = 0.749) has the poorest sensitivity, yet highest specificity. The sensitivity and specificity of the SNR (cut-off score = −19.3 dB, AUC = 0.677) lie in between the two EmNSA-UE measures. After correction for multiple testing none of the AUCs were significantly different from one another.

## Discussion

We hypothesized that SNR of the evoked cortical response to robotic wrist perturbations would follow the typical recovery pattern in which most improvements occur within ten weeks after stroke. We also hypothesized that SNR would be larger in patients with unimpaired proprioceptive function than patients with impaired proprioception. Lastly, we hypothesized that SNR could predict motor recovery at 26 weeks after stroke more accurately than EmNSA-UE.

In contrast to our first hypothesis our results did not reveal any significant effect of time after stroke on SNR. In line with our second hypothesis SNR is significantly larger in patients with unimpaired proprioception, measured with the EmNSA-UE, as compared to patients with impaired proprioception within three weeks after stroke. Lastly, in contrast to our third hypothesis the ability to predict motor function at 26 weeks after stroke, as specified by the area under the ROC curve, did not differ significantly between SNR and the proprioceptive subsection and the total score of the EmNSA-UE. These findings suggest that the magnitude of the evoked cortical response to robotic wrist perturbations remains invariant during the first 26 weeks after stroke and seems to reflect the level of proprioceptive impairment irrespective of time after stroke. Furthermore, the predictive value of the evoked cortical response to predict motor function at 26 weeks after stroke seems to be similar to the predictive value of the EmNSA-UE.

The absence of an effect of time after stroke on SNR is uncommon as measures of neurological and motor function typically change as a function of time after stroke, where most improvement takes place in the first ten weeks after stroke (van der Vliet et al., 2020). Although a thorough explanation for this absence of a time effect cannot be provided based on the presented data, a possible explanation of our finding may be that the evoked cortical response is not affected by mechanisms of spontaneous neurobiological recovery. Alternatively, the evoked cortical response may rather reflect persistent neurological damage in the brain caused by the stroke. As the robotic perturbation continuously manipulated the wrist angle, we argue that the perturbation mainly stimulated muscle spindles in the extensor and flexor muscles of the wrist, thereby stimulating proprioceptive pathways that convey information about the wrist movements to the brain. The SNR of the evoked cortical response to robotic wrist perturbations may therefore be a potential early biomarker for persistent damage to ascending proprioceptive pathways and/or the sensory cortex after stroke. This interpretation is also in line with recent studies suggesting that somatosensory deficits are related to lesions in the thalamocortical radiation (Meyer et al., 2016) as well as cortical areas including the primary and secondary somatosensory cortex and the dorsal insular cortex (Kenzie et al., 2019; Kessner et al., 2019).

As spontaneous recovery of somatosensory function is a prerequisite for regaining full motor function after stroke (Vidoni and Boyd, 2009; Zandvliet et al., 2020a), the evoked cortical response to such proprioceptive perturbations may also be used as an early predictor for motor function at 26 weeks after stroke. Indeed, the evoked cortical response had similar predictive value as EmNSA-UE.

This similar predictive value may be promising for future studies. Evidence from previous studies indicates that somatosensory deficits are a prognostic indicator of poor motor recovery (Meyer et al., 2014; Zandvliet et al., 2020a) and explain variation between patients in therapy induced improvements in hand function (Ingemanson et al., 2019). However, clinical scales that are typically used in neurorehabilitation to assess such somatosensory deficits often lack reliability and sensitivity (Findlater and Dukelow, 2017). Furthermore, these assessments are subjective in nature and require a verbal response from the patients, for instance while administering EmNSA-UE. Therefore, there is a need for proprioceptive assessments, which can apply proprioceptive stimuli in a controlled and reliable manner and measure the neural response quantitatively without a verbal response from the patient.

Several robotic systems to assess proprioceptive function have been developed, which provide proprioceptive stimuli in a controlled and reliable manner [see for instance (Cappello et al., 2015; Contu et al., 2017; Rinderknecht et al., 2018)]. The present study proposes the use of SNR to investigate objectively to what extent proprioceptive stimuli may reach the cortex, without the requirement for patients to verbally respond or actively execute a motor or cognitive task. The evoked cortical response may therefore be widely applicable in patients with stroke, even in patients with aphasia, attention deficits or paralysis of the upper extremity.

To date, studies that used EEG in the first few weeks after stroke also investigated cortical activity at level of the electrodes. A recent study used position-cortical coherence (PCC) to quantify the evoked cortical response (Zandvliet et al., 2020b). In line with our findings, in this study, it was concluded that PCC reflects afferent pathway integrity (Zandvliet et al., 2020b). However, where the present study did not show any effect of time on the evoked cortical response, Zandvliet and colleagues observed significant improvements in PCC in the first twelve weeks after stroke, which correlated with improvements in sensory and motor function within patients (Zandvliet et al., 2020b). Another study, that was performed in partially the same sample of patients as in the present study, investigated longitudinal changes in neural oscillations during resting state at electrode level as a function of time post stroke (Saes et al., 2020). In this study, it was found that the ratio between slow and fast oscillations (delta-alpha ratio) as well as the symmetry of slow oscillations between both hemispheres correlated well with motor and neurological function between and within subjects. The within subject correlations found in these studies suggest that PCC and oscillatory measures reflect mechanisms of spontaneous neurobiological recovery. As the evoked cortical response, as quantified by SNR, did not seem to reflect recovery mechanisms but possibly the degree of persistent neurological damage, we suggest that resting state measures, PCC and SNR may be used as complementary measures in future studies.

The present study has some limitations. A relatively large proportion (19 out of 53) of patients dropped out of the study. These dropouts may particularly concern patients with severe loss of sensory and/or motor function. This might explain why only 23 % of the patients in our sample demonstrated some degree of proprioceptive impairment (7 out of 31 patients). Previous studies have indicated that in general about 50% of all patients with stroke suffers from proprioceptive impairments (Carey, 1995; Connell et al., 2008). Therefore, generalization of the present results to an average population of patients with ischemic stroke is limited. Also, due to the small number of patients in our sample the event-per-variable rule was not satisfied (Peduzzi et al., 1996). This may explain why no significant differences were found between the predictive values of the evoked cortical response and the clinical somatosensory assessments.

Another limitation is that the spatial resolution of EEG is low. EEG is a technique which records electrical potentials generated by electrical sources in the brain, i.e., ionic currents within pyramidal neurons in the cortex. The neuronal electrical currents originating from multiple sources in response to the robotic perturbations may induce electrical potentials in a wide region of the scalp as a result of volume conduction (Buzsáki et al., 2012). Therefore, we had to select a large region-of-interest of fifteen electrodes from which we determined SNR. Consequently, within- and between-subject differences in the location of the evoked cortical response could not be captured with the method presented in this study. To further assess whether the evoked cortical response to robotic perturbations could be used as a biomarker for integrity of proprioceptive pathways, future studies should include a larger cohort of patients in which severely affected patients with initial proprioceptive disorders are well represented. In addition, these studies could use source localization techniques (Nunez, 1974; Michel and Brunet, 2019) to identify how the location of the evoked cortical response may differ between patients with impaired and unpaired proprioception. Such between-subject differences may reveal cortical regions which are critical for proprioceptive function. Moreover, source localization techniques may reveal whether the location of the evoked response changes as a function of time after stroke. Such within-subject changes may indicate whether and how cortical areas may compensate for lost neuronal function after stroke.

If future studies are able to show the complementary value of evoked cortical responses for the prediction of motor recovery after stroke with respect to existing clinical scales, in particular in patients with severe somatosensory deficits, it may then complement current clinical predictors such as the EmNSA-UE. As such, evoked cortical responses may ultimately contribute to more accurate prediction of motor function in patients with a severe paresis at stroke onset.

## Conclusion and Implications

In the present study we conclude that the evoked cortical response to robotic wrist perturbations, as measured by the SNR, seems to reflect sustained proprioceptive impairment of the paretic upper extremity after stroke due to persistent structural damage. Furthermore, SNR has similar predictive value for the prediction of motor function in the paretic upper extremity after stroke as compared to EmNSA-UE. Hence, in the future the evoked cortical response may be used as a biomarker for proprioceptive integrity in clinical practice and may improve prognosis in individual patients early after stroke.

## Members of the 4D-EEG Consortium

In addition to the authors of the present study, the 4D-EEG consortium consists of Jan de Munck, Mique Saes^∗^, Luuk Haring^∗^, Caroline Winters^∗^, Aukje Andringa^∗^, Dirk Hoevenaars^∗^, Ines de Castro Fernandes^∗^, and Sarah Zandvliet^∗^ from Amsterdam University Medical Center; Andreas Daffertshofer from VU University Amsterdam; Yuan Yang, Jun Yao and Julius Dewald from Northwestern University; Dianna Makkus^∗^, and Margot Simonis^∗^ from Reinier de Graaf Hospital; Yvonne Greeuw^∗^, Martijn Vlaar, Konstantina Kalogianni, and Lena Filatova from Delft University of Technology.

^∗^These consortium members performed the measurements.

## Data Availability Statement

The raw data supporting the conclusions of this article will be made available by the authors, without undue reservation.

## Ethics Statement

The studies involving human participants were reviewed and approved by Medical Ethical Reviewing Committee of the Amsterdam University Medical Center, location VU University Medical Center. The patients/participants provided their written informed consent to participate in this study.

## Author Contributions

GK and FH initiated the 4D-EEG project in which the present study was embedded. CM, EW, AS, GK, and FH conceived and designed the experiment. JK was member of the data acquisition team, conducted data analysis, and wrote the manuscript. MR and TS-E conducted data analysis. LA was responsible for patient recruitment. All authors reviewed the manuscript and approved the submitted version.

## Conflict of Interest

The authors declare that the research was conducted in the absence of any commercial or financial relationships that could be construed as a potential conflict of interest.

## Publisher’s Note

All claims expressed in this article are solely those of the authors and do not necessarily represent those of their affiliated organizations, or those of the publisher, the editors and the reviewers. Any product that may be evaluated in this article, or claim that may be made by its manufacturer, is not guaranteed or endorsed by the publisher.
