# Supplementary material for: The Cortical Response Evoked by Robotic Wrist Perturbations Reflects Level of Proprioceptive Impairment After Stroke
Source: Front Hum Neurosci. 2021 Nov 9;15:695366. doi: 10.3389/fnhum.2021.695366 (PMC8631193; doi:10.3389/fnhum.2021.695366)
Supplement: Supplementary file 1 [file Table_1.DOCX]

| **Supplementary table** Sex, age, EmNSA-UE_p_, EmNSA-UE_t_, FM-UE and SNR for each patient and each measurement | | | | | | | | | | | | | | | | | | | | | | |
| --- | --- | --- | --- | --- | --- | --- | --- | --- | --- | --- | --- | --- | --- | --- | --- | --- | --- | --- | --- | --- | --- | --- |
| **Patient number** | **Sex (F/M)** | **Age (y)** | **EmNSA-UE_p_ (points)** | | | | | **EmNSA-UE_t_ (points)** | | | | | **FM-UE (points)** | | | | | **SNR (dB)** | | | | |
|  |  |  | **w1** | **w3** | **w5** | **w12** | **w26** | **w1** | **w3** | **w5** | **w12** | **w26** | **w1** | **w3** | **w5** | **w12** | **w26** | **w1** | **w3** | **w5** | **w12** | **w26** |
| **Impaired proprioception** | | |  |  |  |  |  |  |  |  |  |  |  |  |  |  |  |  |  |  |  |  |
| 1 | M | 64 |  | 3 | 4 | 3 | 6 |  | 6 | 20 | 19 | 22 |  | 5 | 5 | 10 | 10 |  | -21,27 | -20,29 | -20,46 | -21,02 |
| 2 | F | 71 |  | 1 | 2 | 4 | 4 |  | 1 | 2 | 12 | 9 |  | 5 | 6 | 6 | 6 |  | -20,86 | -20,10 | -20,10 | -21,39 |
| 3 | M | 79 | 7 | 8 | 8 | 8 | 8 | 29 | 32 | 36 | 33 | 38 | 34 | 43 | 55 | 58 | 57 |  | -19,20 | -20,87 | -19,85 | -21,70 |
| 4 | M | 78 |  | 1 | 0 | 0 | 0 |  | 1 | 0 | 2 | 1 |  | 24 | 40 | 36 | 42 |  | -20,70 | -19,53 | -19,88 | -19,04 |
| 5 | M | 56 |  | 5 | 6 | 8 | 8 |  | 13 | 20 | 31 | 32 |  | 13 | 16 | 36 | 40 |  | -22,25 | -20,50 | -20,66 | -19,37 |
| 6 | M | 72 |  | 7 | 8 | 8 | 8 |  | 25 | 33 | 39 | 38 |  | 50 | 54 | 59 | 60 |  | -13,51 | -11,02 | -15,00 | -10,79 |
| 7 | M | 80 | 2 |  | 2 | 8 | 8 | 4 |  | 6 | 20 | 18 | 8 |  | 17 | 14 | 18 | -22,47 |  | -21,06 | -20,07 | -21,88 |
|  |  |  |  |  |  |  |  |  |  |  |  |  |  |  |  |  |  |  |  |  |  |  |
| **Unimpaired proprioception** | | |  |  |  |  |  |  |  |  |  |  |  |  |  |  |  |  |  |  |  |  |
| 8 | M | 73 |  | 8 | 8 | 8 | 8 |  | 40 | 40 | 40 | 40 |  | 56 | 59 | 60 | 60 |  | -19,23 | -20,82 | -19,02 | -18,50 |
| 9 | F | 86 | 8 | 8 | 8 | 8 | 8 | 40 | 38 | 40 | 40 | 40 | 36 | 60 | 62 | 62 | 63 | -16,24 |  | -15,19 | -15,84 | -14,58 |
| 10 | F | 76 |  | 8 | 8 | 8 | 8 |  | 37 | 39 | 40 | 40 |  | 11 | 33 | 50 | 59 |  | -14,54 | -14,95 | -16,77 | -14,58 |
| 11 | F | 52 |  | 8 | 8 | 8 | 8 |  | 38 | 38 | 40 | 40 |  | 5 | 7 | 11 | 25 |  | -14,65 | -15,27 | -14,29 | -12,23 |
| 12 | M | 58 |  | 8 | 8 | 8 | 8 |  | 37 | 37 | 39 | 39 |  | 20 | 25 | 39 | 51 |  | -15,85 | -16,26 | -15,05 | -14,18 |
| 13 | F | 72 |  | 8 | 7 | 8 | 8 |  | 35 | 39 | 40 | 40 |  | 6 | 6 | 9 | 15 |  | -17,54 | -15,77 | -15,05 | -13,91 |
| 14 | F | 93 |  | 8 | 8 | 8 | 8 |  | 34 | 33 | 38 | 36 |  | 7 | 10 | 10 | 10 |  | -16,11 | -15,87 | -15,32 | -16,44 |
| 15 | M | 77 |  | 8 | 8 | 8 | 8 |  | 35 | 40 | 38 | 40 |  | 49 | 60 | 62 | 63 |  | -17,93 | -17,55 | -16,23 | -17,36 |
| 16 | F | 70 | 8 | 8 | 8 | 8 | 8 | 36 | 36 | 38 | 38 | 38 | 48 | 52 | 59 | 64 | 64 |  | -18,17 | -17,82 | -17,76 | -18,77 |
| 17 | F | 75 |  | 8 | 8 | 8 | 8 |  | 38 | 36 | 39 | 39 | 42 | 59 | 61 | 63 | 64 |  | -17,75 | -17,00 | -16,51 | -17,64 |
| 18 | M | 63 | 8 |  | 8 | 8 | 8 | 40 |  | 40 | 40 | 40 | 39 |  | 62 | 62 | 64 |  | -16,73 | -15,00 | -13,58 | -15,42 |
| 19 | F | 94 |  | 8 | 8 | 8 | 8 |  | 39 | 40 | 40 | 39 |  | 9 | 12 | 13 | 10 |  | -13,55 | -14,03 | -12,39 | -13,42 |
| 20 | M | 54 | 8 | 8 | 8 | 8 | 8 | 40 | 40 | 40 | 40 | 40 | 15 | 33 | 44 | 59 | 62 |  | -16,17 | -16,29 | -16,00 | -15,99 |
| 21* | F | 85 |  | 8 | 8 | 8 | 8 |  | 40 | 36 | 38 | 36 |  | 59 | 62 | 64 | 64 |  | -16,34 | -18,16 | -16,59 |  |
| 22 | F | 70 | 8 | 8 | 8 | 8 | 8 | 36 | 40 | 40 | 40 | 40 | 31 | 57 | 64 | 62 | 65 |  | -15,11 | -15,83 | -16,33 | -15,13 |
| 23 | M | 74 | 8 | 8 | 8 | 8 | 8 | 40 | 39 | 40 | 39 | 40 | 9 | 37 |  |  | 45 |  | -19,44 | -19,16 | -19,97 | -19,75 |
| 24 | M | 43 | 8 | 8 | 8 | 8 | 8 | 39 | 40 | 40 | 40 | 40 | 8 | 21 | 24 | 44 | 58 |  | -15,93 | -17,81 | -19,50 | -17,71 |
| 25* | M | 77 |  | 8 | 8 | 8 | 8 |  | 40 | 40 | 40 | 40 |  | 7 | 7 | 19 | 24 |  | -18,59 | -17,67 | -18,68 |  |
| 26 | F | 78 |  | 8 | 8 | 8 | 8 |  | 40 | 40 | 38 | 37 |  | 59 | 63 | 63 | 63 |  | -16,10 | -17,35 | 0,00 | -16,84 |
| 27 | M | 59 |  | 8 | 8 | 8 | 8 |  | 40 | 40 | 40 | 40 |  | 7 | 5 | 6 | 7 |  | -19,39 | -20,61 | -20,57 | -19,46 |
| 28 | M | 62 |  | 8 | 8 | 8 | 8 |  | 40 | 40 | 40 | 40 |  | 27 | 49 | 56 | 60 |  | -12,16 | -12,19 | -11,45 | -13,75 |
| 29 | M | 58 | 8 | 8 | 8 | 8 | 8 | 40 | 40 | 40 | 40 | 40 | 6 | 38 | 53 | 61 | 63 | -21,80 | -17,21 | -15,03 | -17,76 | -16,57 |
| 30 | F | 64 | 8 |  | 8 |  | 8 | 40 |  | 40 |  | 39 | 6 |  | 24 |  | 38 | -13,43 |  | -13,48 | 0,00 | -14,96 |
| 31 | M | 62 | 8 |  | 8 | 8 | 8 | 37 |  | 40 | 40 | 39 | 59 |  | 64 | 65 | 63 | -16,32 |  | -18,51 | -17,52 | -17,41 |
| 32 | M | 71 | 8 |  | 8 | 8 | 8 | 36 |  | 36 | 38 | 40 | 49 |  | 58 | 65 | 64 | -16,60 |  | -18,30 | -17,12 | -15,43 |
| 33* | F | 85 |  |  | 8 | 8 | 8 |  |  | 39 | 40 | 40 | 5 |  | 48 | 61 | 48 | -16,99 |  |  | -12,59 |  |
| 34 | M | 69 | 8 |  | 8 | 8 | 8 | 36 |  | 39 | 40 | 39 | 65 |  | 66 | 66 | 66 | -18,12 |  | -18,57 | -16,83 | -18,41 |
| Abbreviations: **dB** decibel **EmNSA-UE_p_** proprioceptive subscore of the upper extremity section of the ErasmusMC modified Nottingham Sensory Assessment **EmNSA-UE_t_** total score of the upper extremity section of the ErasmusMC modified Nottingham Sensory Assessment **F** female **FM-UE** upper extremity section of the Fugl-Meyer Motor Assessment **M** male **w** week **SNR** signal-to-noise ratio **y** years  * Data from these patients were excluded for the analysis corresponding to research question 1 and 2 and were only included for analysis corresponding to research question 3. | | | | | | | | | | | | | | | | | | | | | | |
